# Simultaneous subchronic exposure to selenium and diazinon as possible risk factor for osteoporosis in adult male rats

**DOI:** 10.1186/1751-0147-55-81

**Published:** 2013-11-17

**Authors:** Monika Martiniaková, Ivana Boboňová, Radoslav Omelka, Birgit Grosskopf, Hana Chovancová, Jana Španková, Róbert Toman

**Affiliations:** 1 Department of Zoology and Anthropology, Constantine the Philosopher University, 949 74, Nitra, Slovakia; 2 Department of Botany and Genetics, Constantine the Philosopher University, 949 74, Nitra, Slovakia; 3 Institute of Zoology and Anthropology, Georg-August University, 37 073, Göttingen, Germany; 4 Department of Veterinary Sciences, Slovak University of Agriculture, 949 76, Nitra, Slovakia

## Abstract

**Background:**

Osteoporosis and its main health outcome, fragility fractures, are large and escalating health problems. Skeletal damage may be the critical result of low-level prolonged exposure to several xenobiotics in the general population, but the mechanisms of their adverse effects are not clearly understood. The current study was aimed to investigate the possible ability of simultaneous subchronic peroral administration of selenium (Se) and diazinon (DZN) to induce changes in bone of adult male rats.

In our study, twenty 1-month-old male Wistar rats were randomly divided into two experimental groups. In the first group, young males were exposed to 5 mg Na_2_SeO_3_/L and 40 mg of DZN/L in drinking water, for 90 days. Ten 1-month-old males without Se and DZN intoxication served as a control group. At the end of the experiment, macroscopic and microscopic structures of the femurs were analysed using analytical scales, sliding instrument, and polarized light microscopy.

**Results:**

The body weight, femoral length and cortical bone thickness were significantly decreased in rats simultaneously exposed to Se and DZN (*P* < 0.05). These rats also displayed different microstructure in the middle part of the compact bone where vascular canals expanded into central area of *substantia compacta*. The canals occurred only near endosteal surfaces in rats from the control group. Additionally, a smaller number of primary and secondary osteons, as well as a few resorption lacunae were observed near endosteal surfaces in rats simultaneously administered to Se and DZN. The resorption lacunae as typical structures of bone resorption manifestation are connected with an early stage of osteoporosis. Histomorphometric analysis revealed that area, perimeter, maximum and minimum diameters of primary osteons’ vascular canals were significantly increased (*P* < 0.05) in the Se-DZN-exposed rats. On the other hand, all measured variables of Haversian canals and secondary osteons were considerable reduced (*P* < 0.05) in these rats.

**Conclusions:**

Simultaneous subchronic peroral exposure to Se and DZN induces changes in macroscopic and microscopic structures of the femurs in adult male rats, and also it can be considered as possible risk factor for osteoporosis. The current study contributes to the knowledge on damaging impact of several xenobiotics on the bone.

## Background

Bone is a dynamic mineralized connective tissue constantly being remodelled. Bone growth, mineralization and remodeling are regulated by a complex array of feedback mechanisms depending on age, genetic, nutritional and environmental factors [1-3]. Toxicological studies have shown that bone metabolism is highly sensitive to environmental pollutants (i.e., heavy metals, pesticides) which can alter bone composition and mineralization, producing specific bone pathologies [4-6]. However, these environmental exposures have only been studied to a limited extent.

Selenium (Se) is an essential trace element which occurs in various concentrations in the soil, water leading to variable Se contents in food [7]. Industry utilizes Se in the manufacture of pigments used in variety of applications and pesticide/insecticide formulations. The importance of this element for bone metabolism is still unclear. Several reports are available for deficiency of Se in relation to growth retardation [8,9] and Kashin-Beck disease - a chronic endemic degenerative osteoarthritis [10-12]. On the other hand, excess of Se induces apoptosis in mature osteoclasts [13], osteoblasts [14] and osteoblast-like cells [15]. Furthermore, Se in higher concentration causes abnormal bone and cartilage development and it is reported to be teratogenic [16].

Organophosphorus (OP) compounds are one of the most common types of organic pollutants found in the environment [17]. Residual amounts of OP pesticides have been detected in the soil, water, vegetables, grains and other food products [18]. Diazinon (DZN) is an organophosphate insecticide which acts by inhibiting acetylcholinesterase (AChE) [19]. AChE is most commonly known for its role in terminating cholinergic signalling by the hydrolysis of acetylcholine to choline and acetate. However, recent evidence suggests that AChE may also have a functional role in the bone [4,20-22]. Several skeletal deformities, such as an undulatin notochord and fused cervical rings induced by OP pesticides including DZN have been observed in the study by Misawa *et al*. [23]. Finally, Lari *et al*. [24] revealed highly reduced bone density in rats after four-week treatment by DZN (30 mg/kg per day in corn oil).

Human and animal exposures to several xenobiotics in the environment do not occur in isolation, and also pharmacological agents, other toxins, and diet can induce or supress their toxicity.

Protective effects of Se (due to antioxidant and metal-chelating efficacy) against DZN-induced histopathological changes in various organs have been noted in many studies [25-28], using different (including toxic) doses as well as different types of Se administration. These studies have raised new possibilities for the use of Se against the harmful effects of DZN and potentially also other OP pesticides in practice. However, there is still limited knowledge about possible interactions between DZN and Se in many organs including the bone. Generally, the bone is metabolically very active organ, which accumulates various risk elements and usually is exposed to a relatively long time.

Based on known effects of DZN and Se on the bone and other organs already mentioned above we focused on detailed structural analysis of exposed bones in animal model. Therefore, the aim of our study was to determine in detail the effect of simultaneous subchronic peroral administration of Se and DZN on macroscopic and microscopic structure of femoral bone in adult male rats.

## Methods

### Animals

Twenty 1-month-old male Wistar rats were obtained from the accredited experimental laboratory (number SK PC 50004) of the Slovak University of Agriculture in Nitra (Slovakia). These clinically healthy rats were randomly divided into two experimental groups of 10 individuals. Male rats were used, as they are less susceptible than females to xenobiotics’ toxicity [29-31].

The rats were housed individually in plastic cages in an environment maintained at 20–24°C, 55 ± 10% humidity. They had access to water and food (feed mixture M3, Bonargo, Czech Republic) *ad libitum*. The first group (n = 10 rats) was daily exposed to 5 mg Na_2_SeO_3_/L (98% purity, Reachem, Slovakia) and 40 mg of DZN/L (99% purity, Sigma-Aldrich, USA) in their drinking water for a total of 90 days. The doses of Se and DZN were chosen on the basis of studied literature [32-34] and our previous experiments [35,36] with tested dose–response relationships. The doses were high enough to reach toxicity but also safe enough to prevent animal mortality (non-lethal doses). The dose of Se might be potentially the minimum lethal dose level for adolescent rats for the given route of administration [37]. The second group (n = 10 rats), without Se and DZN exposure, served as the control group. This study was approved by the Animal Experimental Committee of the Slovak Republic.

### Procedures

At the end of 90 days, all the rats were euthanized, weighed and their femurs were used for macroscopic and microscopic analyses. The right femurs were weighed on analytical scales with an accuracy of 0.01 g and the femoral length was measured with a sliding instrument. For histomorphometric analysis, the right femurs were sectioned at the midshaft of the diaphysis and the segments were fixed in HistoChoice fixative (Amresco, USA). The segments were then dehydrated in increasing grades (40 to 100%) of ethanol and embedded in Biodur epoxy resin (Günter von Hagens, Heidelberg, Germany) according to the method described by Martiniaková *et al*. [38]. Transverse thin sections (70–80 μm) were prepared with a sawing microtome (Leitz 1600, Leica, Wetzlar, Germany) and fixed onto glass slides by Eukitt (Merck, Darmstadt, Germany) as previously described [39]. The qualitative histological characteristics of the compact bone tissue were determined according to the internationally accepted classification systems of Enlow and Brown [40] and Ricqlés *et al*. [41], who classified bone tissue into three main categories: primary vascular tissue, non-vascular tissue and Haversian bone tissue. Various patterns of vascularization can occur in primary vascular bone tissue: longitudinal, radial, reticular, plexiform, laminar, lepidosteoid, acellular, fibriform and protohaversian. There are three subcategories indentified in Haversian bone tissue: irregular, endosteal and dense. The quantitative (histomorphometric) variables were assessed using the software Motic Images Plus 2.0 ML (Motic China Group Co., Ltd.). We measured area, perimeter and the minimum and maximum diameters of 424 primary osteons’ vascular canals, 410 Haversian canals and 410 secondary osteons in all views (i.e., anterior, posterior, medial and lateral) of the thin sections in order to minimize inter-animal differences. Diaphyseal cortical bone thickness was also measured by Motic Images Plus 2.0 ML software. Twenty random areas were selected, and average thickness was calculated for each femur.

### Statistics

Statistical analysis was performed using SPSS 8.0 software. All data were expressed as mean ± standard deviation (SD). The unpaired Student’s t-test was used for establishing statistical significance (*P* < 0.05) between both experimental groups.

## Results

### Macroscopic differences

Body weight and femoral length were significantly decreased in rats simultaneously exposed to Se and DZN (*P* < 0.05) in comparison with the control group. Also, cortical bone thickness was significantly lower (*P* < 0.05) in these rats. On the contrary, femoral weight did not differ between the two groups (Table 1).

**Table 1:** **Body weight, femoral weight, femoral length and cortical bone thickness in adult male rats subchronic exposed to 5 mg of Na**_**2**_**SeO**_**3**_**/L and 40 mg of DZN/L in drinking water for 90 days (Se-DZN group) and the control rats**

**Group**	**N**	**Body weight (g)**	**Femoral weight (g)**	**Femoral length (cm)**	**Cortical bone thickness (mm)**
Control	10	405.0 ± 52.7	1.05 ± 0.17	3.94 ± 0.09	0.572 ± 0.054
Se-DZN	10	360.0 ± 17.2	0.93 ± 0.08	3.75 ± 0.07	0.507 ± 0.049
T-test		*P* < 0.05	NS	*P* < 0.05	*P* < 0.05

N: Number of rats, NS: Non-significant changes.

### Microscopic differences

**Figure 1:**
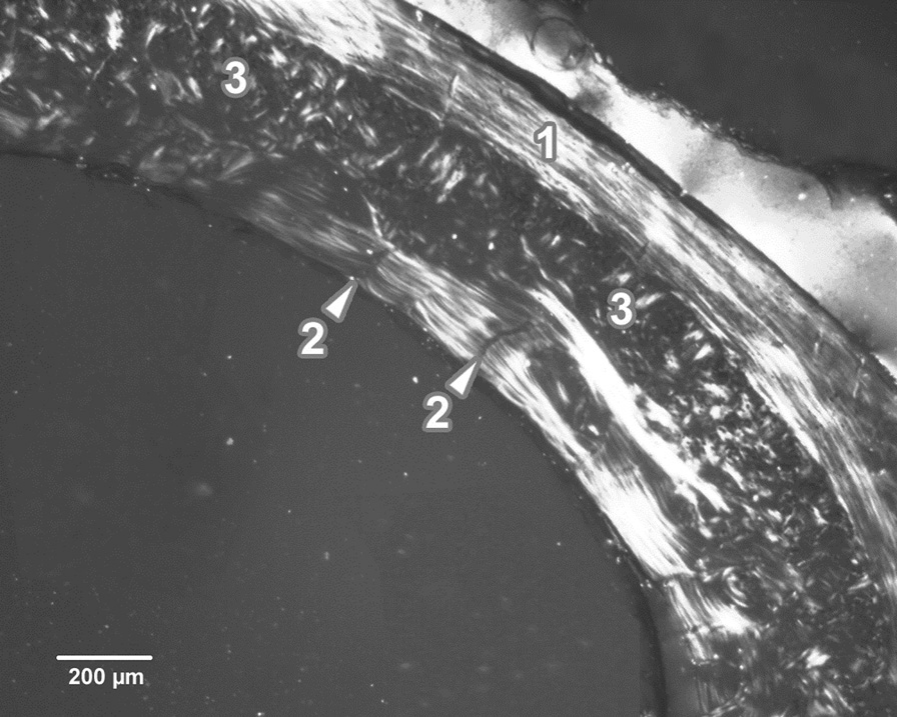
**Microscopic structure of compact bone tissue in rat from the control group (antero-lateral view).** 1. Non-vascular bone tissue. 2. Vascular canals radiating from marrow cavity. 3. Primary and secondary osteons in middle part of compact bone.

Endosteal borders of all femurs from the control rats were formed by non-vascular bone tissue in all views of the thin sections. The bone tissue contained cellular lamellae and osteocytes. Areas of primary vascular radial bone tissue (formed by branching or non-branching vascular canals radiating from the marrow cavity) were also identified in anterior, posterior and lateral views. We found some primary and secondary osteons (especially in the anterior and posterior views) near the endosteal surfaces. In the middle part of the compact bone, primary and secondary osteons were observed. The periosteal border was again composed of non-vascular bone tissue, mainly in the anterior and posterior views (Figure 1).

**Figure 2:**
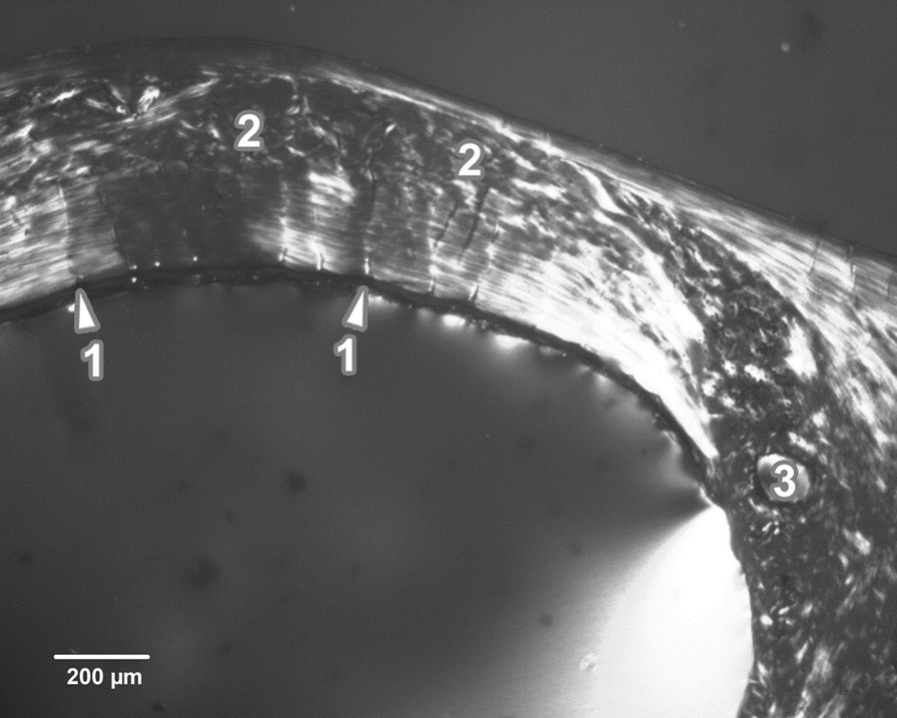
**Microscopic structure of compact bone tissue in rat from the Se and DZN group (antero-lateral view).** 1. Enormous vascular canals radiating from marrow cavity. 2. Smaller number of primary and secondary osteons in middle part of compact bone. 3. Resorption lacunae.

The rats simultaneously exposed to Se and DZN displayed a similar microarchitecture to that of the control rats, except for the middle part of the compact bone in the medial and lateral views. In these views, vascular canals were shown to have expanded into the central area of the bone. The expansion in some cases was so enormous that the canals also occurred near periosteal surfaces. Therefore, a smaller number of primary and secondary osteons was identified in these rats. Moreover, a few resorption lacunae were found near endosteal surfaces in rats co-administered by Se and DZN which indicate the early stage of osteoporosis (Figure 2).

For the quantitative histological analysis, 424 vascular canals of primary osteons, 410 Haversian canals and 410 secondary osteons were measured in total. The results are summarized in Table 2. We found that all measured variables (area, perimeter, maximum and minimum diameters) of the primary osteons’ vascular canals were higher in the Se-DZN-exposed rats than in the control ones (*P* < 0.05). However, these rats displayed significantly decreased levels of all variables of Haversian canals and secondary osteons (*P* < 0.05).

**Table 2:** **Data of the primary osteons’ vascular canals, Haversian canals and secondary osteons in adult male rats subchronic exposed to 5 mg of Na**_**2**_**SeO**_**3**_**/L and 40 mg of DZN/L in drinking water for 90 days (Se-DZN group) and control rats**

	**Group**	**N**	**Area**	**Perimeter**	**Max. diameter**	**Min. diameter**
			**(μm**^**2**^**)**	**(μm)**	**(μm)**	**(μm)**
**Primary osteons’ vascular canals**	Control	218	397.3 ± 98.1	72.29 ± 8.95	12.89 ± 2.08	9.83 ± 1.58
	Se-DZN	206	466.0 ± 107.0	78.96 ± 10.31	14.26 ± 2.64	10.46 ± 1.47
	T-test		*P* < 0.05	*P* < 0.05	*P* < 0.05	*P* < 0.05
**Haversian canals**	Control	208	426.9 ± 119.2	74.47 ± 10.25	13.21 ± 2.16	10.24 ± 1.73
	Se-DZN	202	351.2 ± 69.3	67.54 ± 7.03	11.89 ± 1.69	9.44 ± 1.12
	T-test		*P* < 0.05	*P* < 0.05	*P* < 0.05	*P* < 0.05
**Secondary osteons**	Control	208	6541.0 ± 2012.6	291.79 ± 43.09	52.21 ± 8.61	39.38 ± 7.52
	Se-DZN	202	5623.8 ± 1772.8	268.23 ± 42.00	47.03 ± 8.61	37.49 ± 6.52
	T-test		*P* < 0.05	*P* < 0.05	*P* < 0.05	*P* < 0.05

N: Number of measured structures.

## Discussion

Simultaneous subchronic peroral exposure to 5 mg Na_2_SeO_3_/L and 40 mg of DZN/L in drinking water for 90 days resulted in a significant decrease in body weight and femoral length in adult male rats. Thorlacius-Ussing *et al*. [42] observed growth retardation in rats receiving 15 mg/L Na_2_SeO_3_ in their drinking water which is associated with reduced production of growth hormone (GH) and insulin-like growth factor I (IGF-I). The results by Gronbaek *et al*. [43] also documented a significantly shorter tibia in rats exposed to 3.3 mg Na_2_SeO_3_/L in drinking water for 35 days related to Se-induced significant reduction in circulating IGF-I. In our previous study [36], the decreased body weight and femoral length in rats after application of 5 mg Na_2_SeO_3_/L in their drinking water for 90 days was also observed. DZN is known to show its toxic effects by inhibiting cholinesterase activity. According to Kalender *et al*. [44] and Razavi *et al*. [45] the reduced body weight of rats after DZN supplementation could be caused by less food consumption and/or fluid and electrolyte loss as the result of a reduction in cholinesterase activity. In the study by Ogutcu *et al*. [46], decreased body weight in rats after peroral DZN treatment *via gavage* was identified. DZN-induced inhibition in growth of some skeletal elements, such as femur, tibia, metatarsi and digits of the leg in chick embryos was also demonstrated [23].

The thickness of cortical bone is generally accepted as an important parameter in the evaluation of cortical bone quality and strength. The values of cortical bone thickness in rats from the control group differed from those reported by Comelekoglu *et al*. [47] and Chovancová *et al*. [48], who analysed animals of different age. Statistical analysis has shown significantly decreased cortical bone thickness in rats simultaneously exposed to Se and DZN. The same situation was also observed in rats administered with single dose of 5 mg Na_2_SeO_3_/L in their drinking water for 90 days [31]. Moreover, recalculating and comparing our results with the ones of Martiniaková *et al*. [36], demonstrable differences in cortical bone thickness between the rats exposed to Se in a single dose and those simultaneously intoxicated with Se and DZN were revealed. The Se-DZN-treated rats namely disposed decreased thickness of cortical bone (P < 0.05). This fact suggests a possible synergistic effect of both Se and DZN on bone turnover in rats. Synergistic effect of two (or more) xenobiotics means a combined toxicity that is greater than the simple additive effect of two (or more) compounds [49].

According to Szarek *et al*. [50], Se is able to either increase or decrease toxicity of various xenobiotics (including pesticides) depending on its amounts introduced into an organism. Besides other effects, toxicity of Se occurs due to its prooxidant ability to catalyze the oxidation of thiols and simultaneously generates superoxide [51-53]. Thus, Se is probably able to affect the atom of sulfur (S) in the DZN molecule, and amplifies its toxicity [54]. According to many authors [55-58], Se can substitute S in numerous organic compounds causing their higher reactivity [59]. Similarly to our study, co-administration to Se and DZN induced more significant changes in hepatocyte ultrastructure of rats in comparison with rats administered by Se in single dose [50].

The results of the qualitative histological analysis of the control rats corresponded to those of previous works [60-63]. We identified non-vascular and primary vascular radial tissues and also irregular Haversian bone tissue. However, there was no evidence of true Haversian intracortical bone remodelling. It is generally known that aged rats and mice lack true Haversian cortical bone remodelling but not cancellous bone remodelling [62,64]. Therefore, some secondary osteons can be observed in long bones near the endosteal border. In our study, the newly formed remodelling units within compact bone originated from the endocortical surface and extended deep into the underlying compact bone. The same findings have also been documented in 13 month-old male rats [62].

Simultaneous subchronic exposure to Se and DZN induced changes in the middle part of compact bone where primary vascular radial bone tissue was observed. We proposed that the formation of this type of bone tissue could be explained as an adaptive response to Se and DZN toxicity to protect bone tissue against cell death and subsequent necrosis. The study by Turan *et al*. [65] demonstrates osteocyte loss due to destruction of the bone tissue in rabbits fed excess Se (10 mg Na_2_SeO_3_/kg of diet for a period of 12 weeks). Also, it is known that Se at high doses induces apoptosis in mature osteoclasts [13], osteoblasts [14], and osteoblast-like cells [15]. In respect to osteotoxic effect of DZN, Lari *et al*. [24] reported highly reduced bone density in femoral head, lesser trochanter, greater trochanter and shaft in DZN-exposed rats. In addition, Rangoonwala *et al*. [66] observed a progressive hypocalcemia in rats treated by sublethal dose of DZN. It is generally known that hypocalcemia inhibits calcitonin release. In the absence of this hormone, osteoclast activity is unregulated and bone resorption is accelerated [67]. In our study, accelerated bone resorption was manifested by the presence of resorption lacunae near endosteal surfaces in Se-DZN-exposed rats. These structures are connected with an early stage of osteoporosis. Therefore, intoxication with this mixture of xenobiotics might be one of the reasons for worldwide increasing prevalence of osteoporosis.

Data obtained from the histomorphometric analysis showed a significant increase in area, perimeter, maximum and minimum diameters of the primary osteons’ vascular canals and on the other hand, a significant decrease of the Haversian canals’ variables in the Se-DZN-exposed rats. In general, the vascular system is a critical target for toxic substances and their effects on the vascular system may play an important role in mediating the pathophysiological effects of these substances in specific target organs [68]. Blood vessels readily adapt structurally in response to sustained functional changes, particularly those related to chronic pressure alterations or changes in the nutritional demands of the tissue [69,70]. Results obtained by Cabaj *et al*. [26] demonstrate that the blood vessels of *testes* in rats co-administered by Se and DZN (at the same levels as were used in our study) were damaged and significantly dilated. Additionally, Ruseva *et al*. [71] showed that rats exposed to increased amount of dietary Se had higher glutathione peroxidase 1 (GPx-1) activity and a lower level of anti-elastin antibodies (AEABs) than those of the control group, and the aortic wall histology showed degenerative changes associated with reduced thickness of the wall of the left coronary artery. We also suppose that altered sizes of the primary osteons’ vascular canals and Haversian canals in Se-DZN-intoxicated rats are associated with pathological changes of blood vessels (due to adverse impact of Se and DZN) which are present in both canals. Furthermore, our results indicate that an excess of Se and DZN had a different impact on the primary osteons’ vascular canals and Haversian canals. The main difference between these structures is the presence of a cement line in Haversian canals (cement line delimits the canals) and its absence in primary osteons. We surmise in accordance with our previous study [36] that the cement line could be the main reason for the different results in the histomorphometry of both canals.

We found significantly lower values of all variables of secondary osteons in rats simultaneously exposed to Se and DZN. According to Jowsey [72], the values of secondary osteons are higher in individuals with longer bones. Our results correspond with those found by Jowsey [72], as the femurs were longer in the control rats, which also displayed higher values for osteons. Moreover, we propose that the differences in the size of secondary osteons between Se-DZN-exposed rats and those of control group could be associated with changes in bone remodelling which is mediated by osteoblasts and osteoclasts, and subsequent calcification of bone tissue. Our hypothesis is supported by the results of Chung *et al*. [13] who found that apoptosis in mature osteoclasts could be mediated by Se intoxication. According to Abbott *et al*. [73], decreased osteoclast activity is associated with smaller osteon size. Moreover, Boyar [74] showed that an excess of Se increased the amount of carbonate content in bones of rats injected intraperitoneally with 5 μmol Na_2_SeO_3_/kg for 4 weeks. The incorporation of carbonate ions into the crystal structure of hydroxyapatite (HA) results in changes in the physical and chemical properties of HA [75]. HA crystals, as a major mineral component of bones, are aligned with their long axis parallel to the collagen fibre axis [76] creating concentric lamellae of secondary osteons. Also, it is widely accepted that OP pesticides produce abnormalities in connective tissue [77] through inhibition of the enzyme lysyl oxidase resulting in incomplete post-translational modification of collagen. Secondary osteons contain collagen fibers and therefore, destruction of the collagen could also have an impact on their size.

Our study demonstrates that simultaneous subchronic exposure to Se and DZN had a significant impact on bone structure and causes early stage of osteoporosis in rats. The obtained results can support the better understanding of osteoporosis mechanisms induced by environmental pollutants. However, possible extrapolation of the results to humans may be an interesting topic for discussion. The laboratory rat is preferred animal for most researchers. Its skeleton has been studied extensively, and although there are several limitations to its similarity to the human condition, these can be overcome through detailed knowledge of its specific traits or with certain techniques. The similarities in pathophysiologic responses between the human and rat skeleton, combined with the husbandry and financial advantages, have made the rat a valuable model in osteoporosis research.

## Conclusions

This study demonstrates that simultaneous subchronic peroral administration of 5 mg Na_2_SeO_3_/L and 40 mg DZN/L in drinking water for 90 days affects the body weight, femoral length, cortical bone thickness, and both the qualitative and quantitative histological characteristics of femoral bone tissue in adult male rats. In addition, it induces early stage of osteoporosis. The results can be applied in experimental studies focusing on the effects of various xenobiotics on bone structure, especially when they are considered as possible risk factor for osteoporosis.

## Competing interests

The authors declare that they have no competing interests.

## Authors' contributions

MM was responsible for qualitative histological analysis of bones and writing an article. IB was responsible for quantitative histological analysis of bones. RO was responsible for the statistical analysis. BG was responsible for preparation of histological sections. HC was responsible for macroscopic analysis of bones. JS was responsible for photodocumentation of histological sections. RT was responsible for animal care and sampling of femora. All authors read and approved the final manuscript.
